# Deep sequencing reveals the complex and coordinated transcriptional regulation of genes related to grain quality in rice cultivars

**DOI:** 10.1186/1471-2164-12-190

**Published:** 2011-04-14

**Authors:** RC Venu, MV Sreerekha, Kan Nobuta, André Beló, Yuese Ning, Gynheung An, Blake C Meyers, Guo-Liang Wang

**Affiliations:** 1 Department of Plant Pathology, The Ohio State University, Columbus OH-43210, USA; 2 Delaware Biotechnology Institute, University of Delaware, Newark DE-19711, USA; 3 Crop Biotech Institute, Kyung Hee University, Youngin 446-701, Republic of Korea; 4 State Key Laboratory for Biology of Plant Diseases and Insect Pests, Institute of Plant Protection, Chinese Academy of Agricultural Sciences, Beijing, 100193, China

## Abstract

**Background:**

Milling yield and eating quality are two important grain quality traits in rice. To identify the genes involved in these two traits, we performed a deep transcriptional analysis of developing seeds using both massively parallel signature sequencing (MPSS) and sequencing-by-synthesis (SBS). Five MPSS and five SBS libraries were constructed from 6-day-old developing seeds of Cypress (high milling yield), LaGrue (low milling yield), Ilpumbyeo (high eating quality), YR15965 (low eating quality), and Nipponbare (control).

**Results:**

The transcriptomes revealed by MPSS and SBS had a high correlation co-efficient (0.81 to 0.90), and about 70% of the transcripts were commonly identified in both types of the libraries. SBS, however, identified 30% more transcripts than MPSS. Among the highly expressed genes in Cypress and Ilpumbyeo, over 100 conserved *cis *regulatory elements were identified. Numerous specifically expressed transcription factor (TF) genes were identified in Cypress (282), LaGrue (312), Ilpumbyeo (363), YR15965 (260), and Nipponbare (357). Many key grain quality-related genes (i.e., genes involved in starch metabolism, aspartate amino acid metabolism, storage and allergenic protein synthesis, and seed maturation) that were expressed at high levels underwent alternative splicing and produced antisense transcripts either in Cypress or Ilpumbyeo. Further, a time course RT-PCR analysis confirmed a higher expression level of genes involved in starch metabolism such as those encoding ADP glucose pyrophosphorylase (AGPase) and granule bound starch synthase I (GBSS I) in Cypress than that in LaGrue during early seed development.

**Conclusion:**

This study represents the most comprehensive analysis of the developing seed transcriptome of rice available to date. Using two high throughput sequencing methods, we identified many differentially expressed genes that may affect milling yield or eating quality in rice. Many of the identified genes are involved in the biosynthesis of starch, aspartate family amino acids, and storage proteins. Some of the differentially expressed genes could be useful for the development of molecular markers if they are located in a known QTL region for milling yield or eating quality in the rice genome. Therefore, our comprehensive and deep survey of the developing seed transcriptome in five rice cultivars has provided a rich genomic resource for further elucidating the molecular basis of grain quality in rice.

## Background

Rice is the staple food crop of more than 50% of the global population, and development of high yielding and high quality rice varieties is essential. Rice grain quality is assessed by its appearance and by its milling, cooking, eating, and nutritional quality [1-3]. Milling yield (the percentage of whole grain remaining after removal of the hulls and bran layers from paddy rice) is a very important characteristic that greatly affects profits for rice farmers. Milling yield or milling efficiency is determined based on the quality of the paddy rice, the milling equipment used and the skill of the mill operator. Milling yield is influenced by grain hardness, chalky area of the grain, grain size and shape, depth of surface ridges, bran thickness, and milling efficiency [4-7]. Agronomic and field managements also affect grain breakage during milling [5,8,9]. Rice eating quality is important because it determines the price of rice in the market. Eating quality is determined by water, protein, starch, and fat content [10-14]. Eating quality is negatively correlated with protein content, stickiness, and hardness of rice [10,11]. The main factors affecting both eating and cooking quality of rice are amylose content, gel consistency and gelatinization temperature [12,13,15,16]. Cooked rice with high amylose content is flaky, dry, hard and non-sticky while rice with low amylose content is sticky, moist, tender and glossy [12,13]. Developing cultivars with high milling yield and eating quality have been the main objectives in rice breeding programs in the last few decades.

Milling yield and eating quality are complex traits controlled by quantitative trait loci (QTLs) [17]. In the last several years, many QTLs for eating quality have been mapped in the rice genome. For example, using chromosome segment substitution lines (CSSLs), Wan et al. [18] identified a total of 25 QTLs for nine eating quality traits. Many QTLs affecting different quality traits are mapped in the same chromosomal regions. Six QTLs are non-environment-specific and could be used for marker-assisted selection in rice quality improvement. Recently, Hao et al. [19] constructed 154 CSSLs for QTL mapping of quality traits. In that study, 10 QTLs for rice appearance traits and eight QTLs concerned with physico-chemical traits were detected. QTLs related to glossiness of cooked rice were identified in different genomic regions in Ilpumbyeo, a high grain quality rice in Korea [20]. The amylose content of rice is governed by the waxy (Wx) locus and mapped to chromosome 6 [21-23]. In contrast to the advances in genetic analysis of eating quality, less progress has been made on the genetic analysis of milling quality because the trait has low heritability and is sensitive to environmental factors [24,25]. Another challenge for milling yield analysis is that many mapping populations for milling yield had varied kernel shape among the individual lines and heterogeneity in grain dimensions confounds the assessment of genetic effects [9,24,26-31]. Recently, a mapping study identified six QTLs responsible for head rice (milling) yield using recombinant inbred lines (RILs) derived from crosses of common parent Cypress (high milling) with RT0034 (low milling) and LaGrue (low milling) [9].

The molecular and biochemical basis of grain quality in cereals have been studied in the last decade, and the biochemical processes and many participating genes in the synthesis of starch [32-34], storage proteins [35-39], and lysine within the aspartate family amino acid pathway [40] have been characterized in rice and other cereals. However, how the expression of these genes is coordinated and regulated during grain filling is still poorly understood. Recently, Tian et al. [41] demonstrated that starch synthesis-related genes form a fine network to control eating and cooking qualities by regulating amylose content, gel consistency, and/or gelatinization temperature, and through genetic modification of any of these starch synthesis-related genes, eating and cooking quality can be improved in rice. The expression of 44 genes participating in three pathways (the synthesis of starch, storage proteins, and lysine) during rice grain filling were examined by RT-PCR in the maternal line 93-11 and in the super-hybrid rice line Liang-You-Pei-Jiu (LYP9) [3]. The analysis revealed diverse yet coordinated expression profiles of genes involved in the three pathways in developing seeds. These unique expression patterns of the quality-related genes may influence the final composition and property of starch, protein, and lysine synthesis in rice seeds.

Tools for whole-genome expression analysis like microarrays, serial analysis of gene expression (SAGE) and massively parallel signature sequencing (MPSS) have been widely used for transcriptome analysis in plants in last 10 years [42]. The sequencing-by-synthesis (SBS) second-generation sequencing method has been recently used for transcriptome analysis in many organisms because of its low cost and large sequencing output [43]. In this study, we used both MPSS and SBS to analyze the transcriptome of the developing rice seeds in five cultivars that differed in milling yield and eating quality. Many differentially expressed novel transcripts and genes involved in the biosynthesis of starch, aspartate family amino acids, and storage proteins were identified. Promoter analysis revealed the presence of hundreds of novel conserved patterns of *cis *regulatory elements in the up-regulated genes and putative co-expressed genes in the rice cultivars with high milling yield and good eating quality. Our comprehensive and deep survey of the developing seed transcriptome in five rice cultivars has provided an excellent starting material for further elucidating the molecular and biochemical basis of milling and eating quality in rice.

## Results

### Characteristics of the MPSS and SBS libraries and their matching to the rice genome and to EST and full-length cDNA databases

Both MPSS and SBS tags are short cDNA tags or digital gene expression tags, which are mainly derived from the 3' regions of a transcript [44]. About 1.0 to 1.3 million 17-base MPSS signatures and about 2.0 to 4.0 million 20-base SBS signatures were obtained in the 10 libraries (Table 1). These signatures were clustered and then processed with reliability and significance filters as described by Meyers et al. [45] (Additional File 1). For comparison of the expression levels across the libraries, the frequency of signatures in the individual libraries was normalized to one million (transcripts per million or TPM) [45]. The number of distinct signatures ranged from 12,000 to 18,000 in the MPSS libraries and from 77,000 to 165,000 in the SBS libraries. The SBS libraries contained two to three times more significant signatures (≥4 TPM) than the MPSS libraries. About 79 to 85% of the MPSS and 89 to 95% of the SBS significant signatures matched to the *japonica *(Nipponbare) genomic sequence (Table 1). The significant MPSS and SBS signatures from all five libraries were classified into seven classes based on their location on the annotated genes according to the method previously described by Meyers et al. [45] (Additional File 2).

**Table 1:** Characteristics of the MPSS and SBS libraries of developing rice seeds

Classification	Cypress (PSC)		LaGrue (PSL)		Ilpumbyeo (PSI)		YR15965 (PSY)		Nipponbare (PSN)	
	
	MPSS	SBS	MPSS	SBS	MPSS	SBS	MPSS	SBS	MPSS	SBS
Number of reads	1,266,713	3,718,464	1,082,099	2,326,663	1,201,584	3,293,394	1,190,250	2,634,791	1,207,914	4,110,241
Distinct signatures	12,660	103,741	18,297	165,129	17,783	83,071	12,379	77,023	16,499	104,531
Significant signatures	10,099	30,571	14,253	36,335	13,971	25,915	10,402	23,302	13,116	34,165
Non-significant signatures	2,561	73,170	4,044	128,794	3,812	57,156	1,977	53,721	3,383	70,366
1-100 TPM	10,705	101,187	16,280	161,929	15,605	80,069	10,449	74,564	14,486	101,894
101-1,000 TPM	1,783	2,312	1,827	2,896	1,985	2,704	1,741	2,226	1,815	2,389
1,001-10,000 TPM	157	228	177	290	183	284	176	218	186	234
>10,000 TPM	15	14	13	14	10	14	13	15	12	14
Total signatures matched to the Nipponbare genome	10,940	22,872	15,813	17,521	15,276	20,032	10,678	18,313	14,378	28,118
Significant signatures matched to the Nipponbare genome	8,855 (80.9%)	21,582 (94.3%)	12,534 (79.2%)	15,514 (88.5%)	12,313 (80.6%)	19,000 (94.8%)	9,118 (85.3%)	17,477 (95.4%)	11,622 (80.8%)	25,777 (91.6%)
Significant signatures specifically identified by either MPSS or SBS	2,359	22,831	3,869	25,951	5,219	17,163	3,216	16,116	3,669	24,718
Significant signatures identified by both MPSS and SBS	7,740 (77% overlap)		10,384 (73% overlap)		8,752 (62% overlap)		7,186 (70% overlap)		9,447 (72% overlap)	

### Correlation of the transcriptomic results generated by the MPSS and SBS technologies

From 62 to 77% of the significant signatures overlapped between the MPSS and SBS libraries (Table 1). Further, we used all the significant signatures in the MPSS and SBS libraries of the same cultivar for Pearson correlation coefficient analysis. The correlation coefficient was low when unfiltered MPSS and SBS data were used (Table 2). Removal of a small fraction of outliers (3-8, < 0.001% of the signatures) increased the correlation coefficient significantly in all five libraries (Table 2). For example, the correlation coefficient between the two YR15965 libraries was increased from 0.58 to 0.90 after removal of only four of 5,757 signatures.

**Table 2:** Correlation of the transcriptome results obtained by the MPSS and SBS technologies

Cultivar	Correlation coefficient using all significant signatures*	Correlation coefficient after removing few outliers
Cypress	0.61	0.85 (removal of 3 out of 5,803 signatures)
LaGrue	0.49	0.83 (removal of 6 out of 6,028 signatures)
Nipponbare	0.53	0.87 (removal of 4 out of 7,815 signatures)
Ilpumbyeo	0.39	0.81 (removal of 8 out of 6,985 signatures)
YR15965	0.58	0.90 (removal of 4 out of 5,757 signatures)

* Only genome matched significant signatures were used.

### Expression patterns of grain quality-related genes in the cultivars with high milling yield and good eating quality

Data mining of the TIGR rice annotated genes (pseudomolecules version 5) identified 338 grain quality-related genes belonging to starch biosynthesis and degradation, seed storage protein synthesis (glutelin, globulin, and prolamins), seed maturation, seed allergen synthesis, seed development, and biosynthesis and degradation of aspartate family amino acids (aspartate, asparagine, threonine, isoleucine, methionine, and lysine). We examined the expression level of these genes in developing rice seeds of the five cultivars (Additional File 3). In both SBS and MPSS libraries, a total of 419 (16 grain-related genes) and 168 genes (3 grain-related genes) were ≥5-fold up- and down-regulated, respectively, in Cypress relative to both LaGrue and Nipponbare (Table 3). Similarly, 518 (8 grain-related genes) and 106 genes (4 grain-related genes) were ≥5-fold up- and down-regulated, respectively, in Ilpumbyeo relative to both YR15965 and Nipponbare (Table 3). The number of 5-fold up- and down-regulated antisense genes, genes with antisense transcripts, and genes encoding transcription factors (TFs) in Cypress (compared to both LaGrue and Nipponbare) and Ilpumbyeo (compared to both YR15965 and Nipponbare) are also listed in Table 3.

**Table 3:** The number of over five fold up- and down-regulated genes in Cypress in comparison to LaGrue and Nipponbare and in Ilpumbyeo in comparison to YR15965 and Nipponbare

Classification of genes	Cypress			Ilpumbyeo		
	
	MPSS	SBS	Common	MPSS	SBS	Common
Total number of up-regulated genes	1536 (44)*	4,030 (70)	419 (16)	2,396 (39)	3,339 (50)	518 (8)
Total number of down regulated genes	1409 (33)	1,373 (14)	168 (3)	514 (11)	2,119 (47)	106 (4)
Total number of up-regulated antisense genes	164 (12)	1,266 (32)	23 (5)	256 (7)	1196 (28)	32 (1)
Total number of down regulated antisense genes	53 (7)	330 (4)	6 (0)	22 (5)	372 (17)	3 (1)
Total number of up-regulated genes with alternate transcripts	174	382	3	322	305	9
Total number of down regulated genes with alternate transcripts	131	95	2	57	195	2
Total number of up-regulated transcription factor genes	125	273	37	182	235	14
Total number of down regulated transcription factor genes	102	90	50	34	51	5

*Inside parenthesis is the number of grain quality-related genes

#### Genes involved in starch metabolism

Many genes involved in starch metabolism showed similar expression patterns in both SBS and MPSS libraries (Table 4 and Additional File 4A). For example, the genes encoding 1,4-α-glucan branching enzyme (Os02g32660), limit dextrinase (Os04g08270), 1,4-α-glucan branching enzyme (Os06g51084), and α-amylase (Os09g29404) were 5-fold up-regulated in Cypress compared to LaGrue and Nipponbare in both SBS and MPSS libraries (Table 4).

**Table 4:** List of grain quality genes with similar expression patterns in both SBS and MPSS libraries and up-regulated over five fold in Cypress (in comparison to LaGrue and Nipponbare) and in Ilpumbyeo (in comparison to YR15965 and Nipponbare)

Up-regulated in Cypress	Family	TIGR Gene ID	TIGR gene name	MPSS signature	SBS signature	MPSS Ratio PSC/PSL	MPSS Ratio PSC/PSN	SBS Ratio PSC01/PSL01	SBS Ratio PSC01/PLN02
	Starch synthesis and degradation	Os04g08270	Limit dextrinase, putative, expressed or alpha-amylase	GATCAGATACTCCTCAC	GATCAAATCTGAACCCATCA	49	16.3	8	8
	Starch degradation	Os09g29404	Alpha-amylase activity	GATCTCCTCCTTTTCCT	GATCTGGAAGCGCGCCATTG	25	25	5	5
	Glutelin	Os02g15070	Glutelin type-B 7 precursor	GATCCATTGCACAAGAG	GATCCAGCCACAAACCAATG	22	11	8	8
	Starch synthesis and degradation	Os06g51084	1,4-alpha-glucan branching enzyme, chloroplast precursor	GATCAAGCAATGAATGC	GATCAACCCATGCTCCACCC	24	24	19	19
	Seed specific	Os03g58480	Seed specific protein Bn15D14A	GATCACATCGTCACAGC	GATCTAGAATCTCCAGAGGG	14	28	7	7
	Starch degradation	Os02g32660	Expressed 1,4-alpha-glucan branching enzyme	GATCATGACTTTCAGCA	GATCACAGAAGACACACTTC	6	24	8	8
	Aspartate biosynthesis and degradation II + asparagine biosynthesis I and degradation I	Os02g14110	Nitrogen compound metabolism	GATCTGTGAATTTGGCA	GATCCAGAGAGAGATGCTAA	66	66	8	8
	Globulin	Os03g46100	Globulin-1 S allele precursor	GATCATCCGCGCGTCGG	GATCGTTTAGTTGGGAGTGG	20	20	8	8
	Seed maturation	Os09g10620	seed maturation protein LEA 4	GATCGAGTTGAGTGTGT	GATCGACTTGTGTGAGTTGT	16	16	8	8

**Up regulated in Ilpumbyeo**	**Family**	**TIGR Gene ID**	**TIGR gene name**	**MPSS signature**	**SBS signature**	**MPSS Ratio PSI/PSY**	**MPSS Ratio PSI/PSN**	**SBS Ratio PSI02/PSY02**	**SBS Ratio PSI02/PSN02**

	Methionine degradation I	Os01g22010	Methionine adenosyltransferase	GATCCCGACTTCACATG	GATCCTCGCGGCCGAAATGG	35	35	14	14
	Isoleucine biosynthesis I	Os03g21080	Acetolactate synthase	GATCGGAGCCTAGTTGC	GATCCAGCACACATTCAAAA	5	5	10	10
	Starch synthesis	Os06g12450	Soluble starch synthase 2-3, chloroplast precursor	GATCTGGAAGTGAAATA	GATCTGGAAGTGAAATATTT	12	12	20	20
	Seed allergenic/lectin	Os07g11510	Seed allergenic protein RA5 precursor	GATCGCCTCGCACCTGC	GATCACTTTAGTCTTTATAG	15	15	24	24
	Starch degradation	Os02g32660	Expressed 1,4-alpha-glucan branching enzyme	GATCAGTGTTTTAAGTT	GATCAAATTACATATTGCTG	24.5	49	56	56
	Starch synthesis	Os06g04200	Granule-bound starch synthase 1, chloroplast precursor	GATCTTCCACAGCAACA	GATCTTGGCAAGTCAATTAA	6	6	14	14
	Isoleucine degradation I	Os02g43720	Enoyl-coA hydratase	GATCGTCTTGAAGGTCT	GATCTACCTCCATGCCTTGA	6.2	25	15	15
	Aspartate biosynthesis and degradation II + asparagine biosynthesis I and degradation I	Os01g55540	Aspartate transaminase	GATCAAGTGGCTTTCAT	GATCGGCAAATACTCCTTAA	34	34	32	32

**Figure 1:**
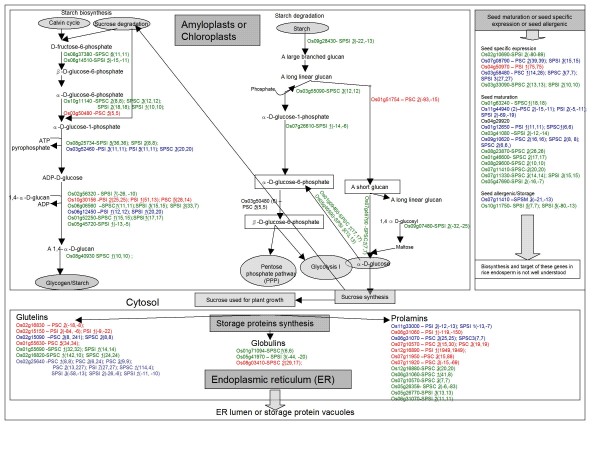
**Network of genes involved in starch biosynthesis and degradation, and in the biosynthesis of seed storage, seed maturation, and allergenic proteins http://www.gramene.org**. Only the genes with 5-fold up- or down-regulation in Cypress (PSC) or Ilpumbyeo (PSI) compared with that in LaGrue or YR15965 are shown. The positive number in parenthesis indicates up-regulation and the negative number in parenthesis indicates down-regulation. The first value in parenthesis shows the fold change in expression either in LaGrue or YR15965, and the second value shows the fold change in expression in Nipponbare. The italicized and underlined bold number before the parenthesis shows the MPSS/SBS signature class [45]. Green indicates that the gene was identified by SBS only. Red indicates that the gene was identified by MPSS only. Blue indicates that the gene was identified by both MPSS and SBS.

Interestingly, we found that genes encoding enzymes involved in the biosynthesis of starch underwent alternative splicing (Figure 1). For example, genes involved in the breakdown of long linear glucan leading to β-D-glucose-6-phosphate (Os03g55090 encoding phosphorylase and Os03g50480 encoding phosphoglucomutase) underwent alternative splicing in Ilpumbyeo and Cypress (Additional File 4A, 4B, 4C). Similarly, the genes encoding the α-amylases (Os09g29404/Os04g08270/Os04g33040/Os01g51754) and 1-4 α- glucan branching enzyme (Os06g51084) involved in the breakdown of short linear glucan leading to β-D-glucose underwent alternative splicing in Ilpumbyeo and Cypress (Additional File 4A, 4B, 4C). Some of the 5-fold up-regulated genes identified by either MPSS or SBS also had alternative splicing forms, and these included genes encoding glucose-1-phosphate adenylyltransferase large subunit 1 (also called AGPase) (Os01g44220) and 1,4-α-glucan branching enzyme (Os06g51084). Similarly, some of the 5-fold down-regulated genes identified by either MPSS or SBS produced alternative splicing forms, and these included genes encoding 1,4-α-glucan branching enzyme (Os06g51084) and phosphoglucomutase (Os03g50480) (Additional File 4A, 4B, 4C). These results showed the complexity of the transcription of quality-related genes in developing rice seeds.

**Figure 2:**
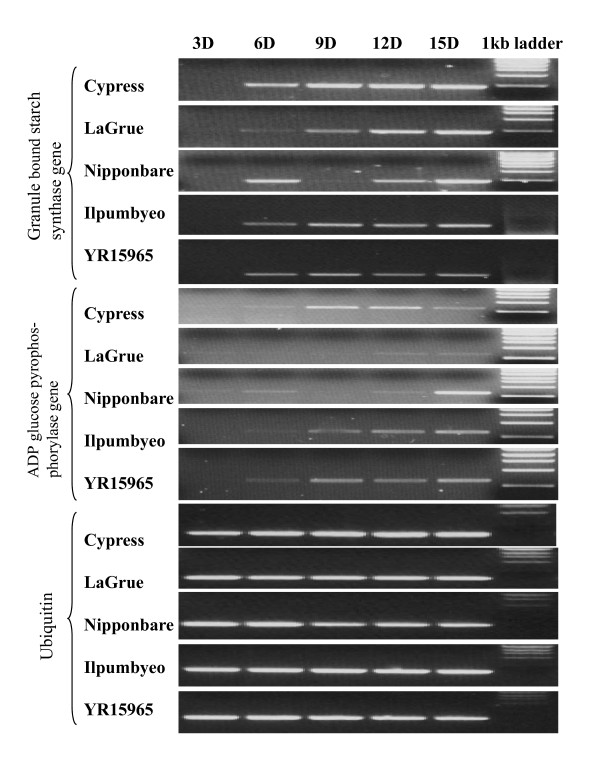
**RT-PCR analysis of the genes encoding GBSS I and AGPase in developing rice seeds at 3, 6, 9, 12, and 15 days after anthesis in five rice cultivars**.

For validation of the MPSS data, two starch biosynthesis-related genes that showed differential expression in the grain libraries were selected for strand specific RT-PCR. These two genes encode AGPase (AK073146) and GBSS I (AK070431). Total RNA was isolated from the developing seeds of Cypress, LaGrue, Ilpumbyeo, YR15965 and Nipponbare at 3, 6, 9, 12 and 15 DAF (days after flowering). A time-course study of the AGPase and GBSS I genes indicated that expression levels were higher in the high milling Cypress than in the low milling LaGrue in the early stages (6 and 9 DAF) of seed development (Figure 2).

#### Genes encoding essential amino acids

The aspartate family pathway consists of five amino acids (asparagine, aspartate, lysine, methionine, and threonine), and is catalysed primarily by the enzymes aspartate kinase (AK) and dihydrodipicolinate synthase (DHPS). The regulatory network of the genes involved in the biosynthesis and degradation of aspartate family amino acids is plotted in Additional File 5. The genes involved in the metabolism of the aspartate family amino acids with 5-fold up- or down-regulation in Cypress and Ilpumbyeo compared to their controls (LaGrue, YR15965, and Nipponbare) are listed in Additional File 4A, 4B, 4C, and Additional File 5). Some of the important genes for amino acid biosynthesis showed similar expression patterns in both MPSS and SBS libraries (Table 4 and Additional File 4A, 4B, 4C). For example, the genes encoding aspartate transaminase (Os01g55540), methionine adenosyltransferase (Os01g22010), and acetolactate synthase (Os03g21080) were 5-fold up-regulated in Ilpumbyeo compared to YR15965 and Nipponbare in both SBS and MPSS libraries. In contrast, some of the genes involved in aspartate family amino acid biosynthesis were down-regulated, including those encoding threonine synthase (Os01g49890), aspartate kinase (Os03g63330), and malate dehydrogenase (Os10g33800) (Additional File 4A, 4B, 4C). In addition, many of the genes involved in the amino acid biosynthesis also underwent alternative splicing. Among them, some showed 5-fold up-regulation in Ilpumbyeo in either the MPSS or SBS libraries, and these included genes encoding L-3-cyanoalanine synthase (Os04g08350), methionine gamma-lyase (Os09g28050), and asparaginase (Os04g46370), which showed two, two, and three alternative splice forms, respectively (Additional File 4A, 4B, 4C).

#### Genes encoding seed-storage proteins

The major classes of storage proteins are glutelins, globulins, and prolamins. Some of the genes encoding these classes showed over 5-fold up-regulation in Cypress compared to LaGrue and Nipponbare, and these genes included those encoding glutelin type-B7 precursors (Os02g15070, Os02g15090), globulin-1 S allele precursor (Os03g46100), prolamin PPROL 17 precursor (Os06g31070), and 13 kDa prolamin precursor (Os07g10570) (Table 4 and Additional File 4A). Among the storage-protein genes with over 5-fold up-regulation in Cypress, some produced antisense transcripts like those encoding glutelin type-B7 precursor (Os02g15070, Os02g15090), prolamin PPROL 17 precursor (Os06g31070), and 13 kDa prolamin precursor (Os07g10570) (Additional File 4A). Many genes encoding glutelins and prolamins also underwent alternative splicing or termination in Cypress and Ilpumbyeo. The gene encoding glutelin type-A 2 (Os02g25640) produced 15 and 17 alternative splice forms in MPSS and SBS libraries, and most of them were up-regulated in Cypress but down-regulated in Ilpumbyeo. Among the prolamin-related genes, the prolamin precursor protein gene (Os07g10570) produced five and six alternative splice forms in MPSS and SBS libraries, respectively. The 5-fold induced or suppressed genes encoding globulin, prolamin, and glutelin storage proteins either in Cypress or Ilpumbyeo or both are listed in Additional File 4A and in Figure 1.

#### Genes encoding seed maturation and allergenic and seed-specific expression proteins

Some of the genes belonging to this group showed similar expression patterns in both MPSS and SBS libraries (Table 4 and Additional File 4A). For example, the genes encoding seed-specific protein Bn15D14A (Os03g58480) and seed-maturation protein LEA4 (Os09g10620) were > 5-fold up-regulated in Cypress compared to LaGrue and Nipponbare in both MPSS and SBS libraries. However, the seed-allergenic protein RA5 precursor gene (Os07g11510) was up-regulated 15-fold in Ilpumbyeo compared to YR15965 (Table 4; Additional File 4A).

### Expression patterns of TF genes in cultivars with high milling and good eating quality

TFs were identified using homology search in the rice TF database http://plntfdb.bio.uni-potsdam.de/v3.0/. Clustering analysis was performed to identify TF genes up- and down-regulated in Cypress and Ilpumbyeo compared to the controls (Table 3; Additional File 6). A total of 37 and 14 TF genes showed 5-fold up-regulation in Cypress and Ilpumbyeo libraries, respectively, in both SBS and MPSS libraries (Additional File 6). Similarly, 50 and 5 TF genes were down-regulated in Cypress and Ilpumbyeo, respectively, in both libraries. Some TFs were specifically up-regulated in either Cypress or Ilpumbyeo compared to the controls in both libraries. These TF genes encode PHD-finger family protein (PHD family; Os01g65600), zinc finger CCCH type domain containing protein ZFN-like 2 (C3H family; Os01g68860), transfactor (G2-like; Os06g40710), and bZIP transcription factor family protein (bZIP family; Os06g45140) (Additional File 6).

### Identification of the conserved *cis *motifs among the up-regulated genes in cultivars with high milling and good eating quality

The promoter sequences (1.0 kb before the ATG site) of the highly up-regulated genes (≥50-fold) in Cypress (compared to LaGrue and Nipponbare) and Ilpumbyeo (compared to YR15965 and Nipponbare) identified in both SBS and MPSS libraries were analyzed using the 'PLACE Signal Scan Search' software http://www.dna.affrc.go.jp/htdocs/PLACE/. Many conserved motifs were present in the up-regulated genes in Cypress and Ilpumbyeo, and these included CAATBOX1, WRKY71OS, GATABOX, EBOXBNNAPA, SEF4MOTIFGM7S, CGACGOSAMY3, WBOXHVISO1, CAREOSREP1, CANBNNAPA, AMYBOX1, AACACOREOSGLUB1, BOXIIPCCHS, 2SSEEDPROTBANAPA, ACGTABOX, AMYBOX2, ACGTCBOX, ACGTOSGLUB1, CEREGLUBOX2PSLEGA, and GADOWNAT (Additional File 7). Interestingly, many of the motifs have been reported to play a role in seed development and germination (Additional File 7) [46-70].

## Discussion

Rice is a major source of nutrition for most people in the developing world. Although tremendous achievements have been made for the improvement of many agronomic traits in rice in the last three decades, much less progress has been obtained for quality traits due to the lack of simple and efficient selection methods in rice breeding. With rapid advancement in crop molecular breeding, marker-aided selection has been successfully applied in many crop plants. Similarly, new methods for genetic engineering of better crop plants have been reported in the last decade by overexpressing or gene silencing of candidate genes. Although several eating quality QTLs have been identified in previous studies [18,19], it is not clear whether these QTLs are useful for marker-aided selection or not because the genomic regions of these QTLs have not been further characterized. Recently Nelson et al. [9] identified six main-effect milling yield QTLs in the two RIL populations derived from crosses of common parent Cypress with RT0034, a low-milling yield japonica line and LaGrue, a low-milling yield japonica cultivar, respectively. In this study, we used two high throughput sequencing technologies to profile the transcriptome of five cultivars differing in milling yield and eating quality. Many genes specifically or commonly expressed in the high milling yield cultivar Cypress and the good eating quality cultivar Ilpumbyeo were identified from the MPSS and SBS libraries. These candidate genes are excellent starting materials for the development of molecular markers linked to milling quality in the US and eating quality in Korea for rice breeding. It is also possible that overexpression or silencing of some candidate genes will lead to the generation of transgenic rice plants with superior grain quality.

During the rice seed development, sugars, amino acids, and other important metabolites are transported from source (primarily leaves) to sink (seeds). Once in the seeds, these metabolites are allocated to different biosynthetic pathways (primarily starch metabolism and storage protein biosynthesis) to produce mainly starch and proteins in precise quantities and ratios. Achieving such a defined composition of starch and proteins require the regulation and coordination of various pathways so that, at each developmental stage, the participating enzymes are present in appropriate amounts and in the correct cellular compartments [3]. AGPase and GBSS I play important roles during starch biosynthesis in rice [71]. The genes encoding for AGPase and GBSS I enzymes are highly expressed 7 to 28 days after flowering during grain development, and their expression is highly correlated with the increases in both starch content and grain weight. The AGPase gene is also highly expressed in the high-yield cultivars of both glutinous and non-glutinous rice [71]. In addition, AGPase (Os01g44220) undergoes alternative splicing similar to the AGPase small subunit gene in barley [72]. Duan and Sun [3] showed that a mutation in the GBSS I gene leads to a lower level of functional GBSS I mRNA and correspondingly to a lower level of GBSS I enzyme for amylose synthesis, which causes a reduction in amylose accumulation. During rice seed formation, the genes encoding AGPases are active 3 days before flowering and maintain an intermediate although declining level of activity during seed maturation [3]. Genetic variation survey showed that the polymorphism in the rice waxy gene encoding the GBSS enzyme explains much of the variation in apparent amylose content across 92 important long, medium and short grain US rice cultivars and 101 progeny of a cross between low-amylose and intermediate-amylose breeding lines [73,74]. The amylose content and the level of waxy protein in 31 rice cultivars from China were correlated with the ability of the cultivar to excise intron I from the leader sequence of the Wx transcript [75]. In this study, we found that the important starch biosynthesis related genes encoding AGPase (Os01g44220), 1,4-α-glucan branching enzyme (Os02g32660), limit dextrinase (Os04g08270), 1,4-α-glucan branching enzyme (Os06g51084), and α-amylase (Os09g29404) were up-regulated in Cypress compared to LaGrue and Nipponbare in six-days old developing seeds. Our time-course RT-PCR analysis also confirmed that expression of AGPase and GBSS I genes was higher in the high milling cultivar Cypress than in the low milling cultivar LaGrue early (6 and 9 DAF) in seed development. These results suggest that these two genes related to starch synthesis may greatly affect milling yield. Starch biosynthesis is also associated with complex genotypic-environmental interactions in maize endosperm [76]. Since the plants in this study were grown in the controlled environmental conditions (growth chambers), the effect of environmental factors on the expression of the starch biosynthesis genes should be tested in the field conditions.

Cereal proteins are generally deficient in lysine, but lysine content might be increased with increased accumulation of the precursor molecules required for the enzymatic reactions involved in lysine metabolism. The key precursor molecules include lactate, acetyl CoA, malate, L-aspartate, L-asparagine, L-aspartate-semialdehyde, homoserine, homocysteine, 2-oxobutanoate, 2-aceto-1-hydroxybutyrate, and α-ketoglutarate, and the enzymes involved in their production are very important (Additional File 5). Enhancing the production of these precursor molecules will require the identification of the genes encoding these enzymes. In this study, we found that the genes encoding malate dehydrogenase (Os03g56280, Os01g46070) and aminotransferase (Os09g28050, Os03g18810) involved in the production of malate and aspartate in Cypress and Ilpumbyeo, respectively, were up-regulated compared to the controls. Genes encoding aspartate transaminase (Os01g55540) and enoyl-CoA hydratase (Os02g43720) enzymes, which are responsible for the production of acetyl CoA, were also up-regulated in Cypress compared to the controls. Similarly, the gene encoding lactoylglutathione lyase (Os05g07940), which is responsible for the production of lactate, was up-regulated in Cypress compared to the controls. As indicated, genetic manipulation of the expression levels of these precursors/enzymes may lead to an increased accumulation of lysine in the endosperm and thus an increased nutritional value of the rice seeds.

In the last decade, oligoarrays, SAGE, MPSS, and SBS have been widely used for transcriptome profiling. MPSS and SBS have been recently used for whole-genome transcription analysis and have generated abundant expression data for many organisms [42,44,45]. In this study, both MPSS and SBS technologies were used to analyze the transcriptomes of the 6-days-old developing seeds in five rice cultivars. The number of redundant and non-redundant signatures generated in this study were similar to those in previous reports in rice and *Arabidopsis *[43,45,77]. Although MPSS generates large volume of data, its complicated library-construction procedure and high sequencing cost limit its use in individual laboratories. As the cost of the next-generation sequencing methods has significantly decreased in the last few years, SBS sequencing has become a popular method for transcriptome analysis because it costs 90% less than MPSS and can generate at least three times more transcripts. Furthermore, in the current study, about 30% more transcripts were found in the SBS library than in the MPSS library. Many of these additional signatures are low-copy transcripts, indicating that SBS is a powerful method for identifying rare transcripts [43]. The correlation coefficient is higher between MPSS and SBS than between RL-SAGE and microarray [78], or between RL-SAGE and MPSS or MPSS and microarrays as in previous studies [79]. Therefore, SBS will undoubtedly become the preferred high throughput sequencing method for deep transcriptome analysis in plants.

## Conclusion

Breeding for milling yield and eating quality in rice has been a daunting task due to the low genetic inheritability of both traits and the lack of molecular markers linked to the phenotypes. Genetic mapping of the two traits is also challenging because the traits are easily affected by environmental factors in the field. Using two high throughput sequencing methods, we identified many differentially expressed genes in developing rice seeds that may affect milling yield or eating quality. Many of the identified genes are involved in the biosynthesis of starch, aspartate family amino acids, and storage proteins. Some of these potential candidate genes could be used for the development of molecular markers for breeding programs or for the engineering of rice cultivars with high milling yield and eating quality. Our study provides a valuable genomic resource for both improvement of rice grain quality and for the characterization of grain quality pathways at the molecular and biochemical levels.

## Methods

### Plant materials, developing seeds harvest and growth conditions

Five rice cultivars including Cypress, LaGrue, Ilpumbyeo, YR15965, and Nipponbare were used in the study. Cypress (*japonica *cultivar) is a long grain cultivar with high yield and high milling quality released by Louisiana State University. Cypress dries down slowly in the field, avoiding grain fissuring, cracking and chalkiness that reduce milling quality http://agebb.missouri.edu/rice/research/99/pg5.htm[80-84]. LaGrue (*japonica *cultivar), a long grain variety released by the University of Arkansas in 1993, has low milling quality [80-84]. Both Cypress and LaGrue seeds were provided by Dr. Robert Fjellstrom, USDA-ARS Dale Bumpers National Rice Research Center, Stuttgart, Arkansas, USA. Ilpumbyeo (*japonica *cultivar) is a good eating quality cultivar with low amylose content [85-87]. YR15965 (*japonica *cultivar) is a low eating quality rice, derived from a cross between Hwayeongbyeo (temperate *japonica *variety) and Shennung 89-366 (sub-tropical *japonica*) [86]. Both Ilpumbyeo and YR15965 seeds were provided by Dr. Gynheung An, Crop Biotech Institute, Kyung Hee University, Korea. Nipponbare (*japonica *cultivar) was used as a control for milling and eating quality with Cypress, LaGrue, Ilpumbyeo and YR15965. All the five cultivars were grown in 3 replications in a Conviron growth chamber at 80% relative humidity with 12 h of light (500 μmol photons m-2 sec-1) at 26°C followed by 12 h of dark at 20°C. The spikelets were labeled on the day of anthesis to identify the age of developing seeds in a panicle. The developing seeds were harvested from the panicles at 3, 6, 9, 12 and 15 D after anthesis. The excised developing seeds from the panicle were freezed immediately in liquid nitrogen.

### RNA isolation and RT-PCR

Total RNA was isolated from developing rice seeds harvested from Cypress, LaGrue, Ilpumbyeo, YR15965 and Nipponbare plants using Trizol reagent (Invitrogen). For removal of polysaccharides/polyglycons from the extract, the extracted RNA was purified twice by high salt precipitation according to the manufacturer's instructions. For the MPSS and SBS library construction, RNA isolated from the 6-days (D)-old developing seeds (intermediate stage of grain filling) was used. For the time-course RT-PCR validation experiments, RNA isolated at 3, 6, 9, 12 and 15 D old developing seeds was used. RT-PCR was performed as described previously [78].

### MPSS and SBS library construction, sequencing, and bioinformatics

MPSS and SBS libraries were constructed using the RNA obtained from 6 days old developing seeds from Cypress (MPSS library-PSC; SBS library-SPSC), LaGrue (MPSS library-PSL; SBS library-SPSL), Ilpumbyeo (MPSS library-PSI; SBS library-SPSI), YR15965 (MPSS library PSY; SBS library-SPSY) and Nipponbare (MPSS library-PSN; SBS library-SPSN). MPSS and SBS library construction and sequencing were performed essentially as previously described [43,45,77]. Data analysis was carried out to identify the genes responsible for milling quality and eating quality. The expression profiles of Cypress were compared with that of LaGrue and Nipponbare to identify the genes responsible for milling quality. Similarly, the expression profiles of Ilpumbyeo were compared with that of YR15965 and Nipponbare to identify the genes responsible for eating quality. Bioinformatic analyses including identification of antisense transcripts, alternate transcripts, and TFs were conducted as previously described [43]. Gramene database http://www.gramene.org was used as a reference database for the identification of genes involved in starch metabolism, aspartate amino acid metabolism, storage and allergenic protein synthesis, and seed maturation [88]. The entire dataset is available at the NCBI's Gene Expression Omnibus (GEO) database through the accession number GSM629225 to GSM629233

## Authors' contributions

RCV and MVS grew the plants, collected developing seeds, extracted RNA and performed RTPCR experiments, constructed SBS libraries, bioinformatic analysis of MPSS and SBS data and wrote the manuscript, KN and AB analyzed the data, GA, BCM and G-LW designed the experimental plan and revised the manuscript. The authors agreed on the contents of the paper.

## Supplementary Material

Additional file 1 **Filter results of the five MPSS and SBS libraries**. A) A total of 39,288 distinct 17-base expressed signatures from the five MPSS libraries were processed according to three filters: significance, reliability, and genomic match. B) Similarly, 397,543 signatures from the five SBS libraries were also processed using these same filters as previously described by Meyers et al. [45]. Click here for file

Additional file 2 **Classification of the MPSS and SBS signatures from the five libraries based on their location on the annotated gene (hits = 1) (See Meyers et al. 2004 **[45]**for details).** Click here for file

Additional file 3 **List of expressed grain quality related genes identified in 6 days old developing seeds by MPSS and SBS technologies**. Click here for file

Additional file 4 **List of five fold up and down-regulated genes, antisense and alternate transcripts**. A: List of genes commonly identified by MPSS and SBS technologies. Five fold up- and down-regulated genes, antisense and alternate transcripts are presented. B: Genes identified by SBS technology. Five fold up- and down-regulated genes, antisense and alternate transcripts are listed. C: Genes identified by MPSS technology. Five fold up- and down-regulated genes, antisense and alternate transcripts are listed. Click here for file

Additional file 5 **Network of lysine and aspartate family amino acid biosynthesis and degradation**. http://www.gramene.org. Only the genes with 5-fold up- or down-regulation in Cypress (PSC) or Ilpumbyeo (PSI) compared with that in LaGrue or YR15965 are shown. The positive number in parenthesis indicates up-regulation and the negative number in parenthesis indicates down-regulation. The first value in parenthesis shows the fold change in expression either in LaGrue or YR15965, and the second value shows the fold change in expression in Nipponbare. The italicized and underlined bold number before the parenthesis shows the MPSS/SBS signature class [45]. Green indicates that the gene was identified by SBS only. Red indicates that the gene was identified by MPSS only. Blue indicates that the gene was identified by both MPSS and SBS. Click here for file

Additional file 6 **Five fold up and down regulated transcription factors identified by MPSS, SBS and both**. Click here for file

Additional file 7 **Conserved *cis *elements in the promoter region of the highly induced genes (≥50 fold) in Cypress (compared to LaGrue and Nipponbare) and Ilpumbyeo (compared to YR15965 and Nipponbare) that are involved in seed development**. Click here for file
