# Supplementary material for: Deep sequencing reveals the complex and coordinated transcriptional regulation of genes related to grain quality in rice cultivars
Source: BMC Genomics. 2011 Apr 14;12:190. doi: 10.1186/1471-2164-12-190 (PMC3098810; doi:10.1186/1471-2164-12-190)
Supplement: Additional file 1 — Filter results of the five MPSS and SBS libraries. A) A total of 39,288 distinct 17-base expressed signatures from the five MPSS libraries were processed according to three filters: significance, reliability, and genomic match. B) Similarly, 397,543 signatures from the five SBS libraries were also processed using these same filters as previously described by Meyers et al. [45]. [file 1471-2164-12-190-S1.PPT]

## Slide 1
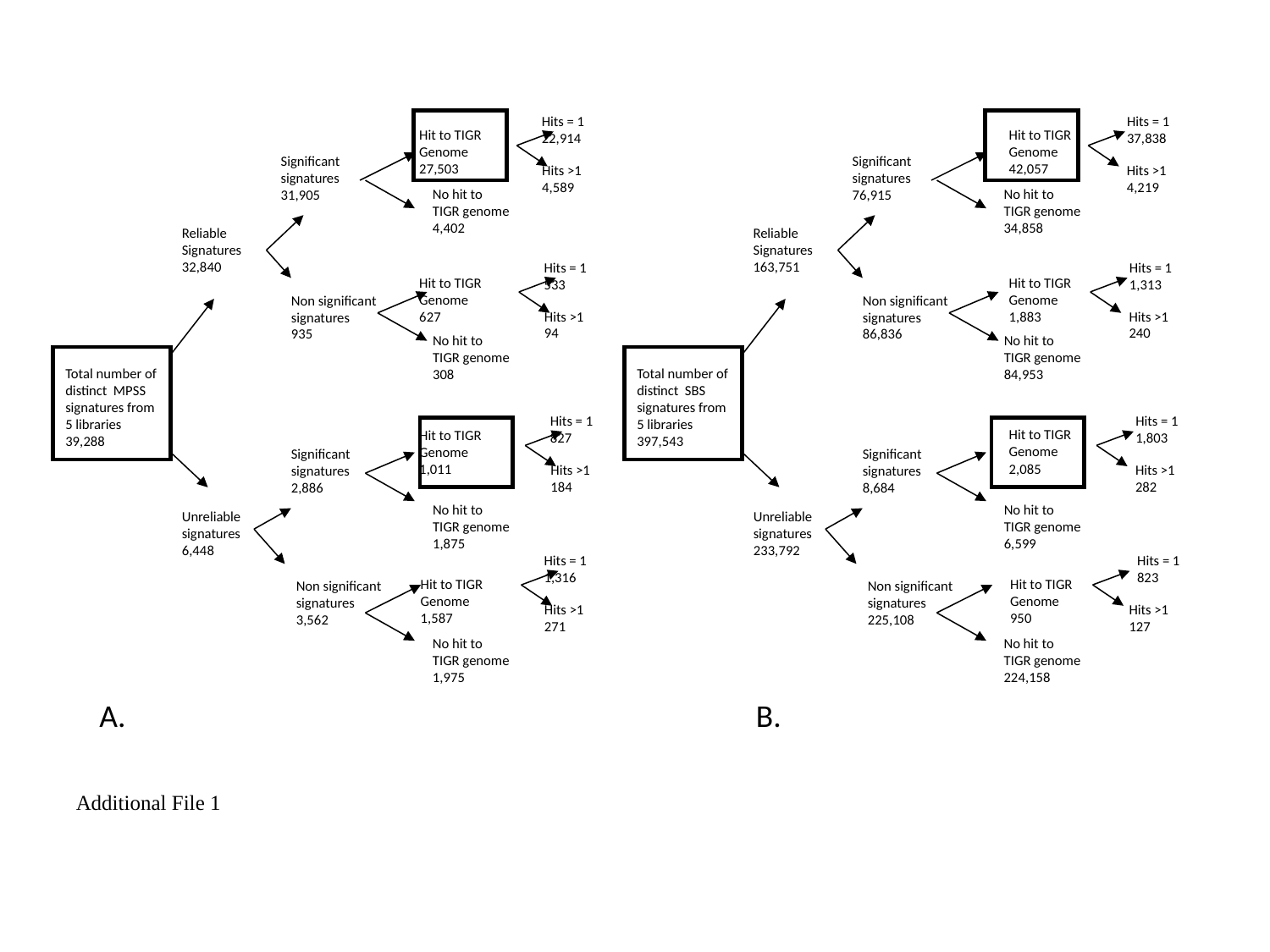

Hits = 1
22,914
Hit to TIGR
Genome
27,503
Significant signatures
31,905
Hits >1
4,589
No hit to TIGR genome
4,402
Reliable
Signatures
32,840
Hits = 1
533
Hit to TIGR
Genome
627
Non significant signatures
935
Hits >1
94
No hit to TIGR genome
308
Total number of distinct MPSS signatures from 5 libraries
39,288
Hits = 1
827
Hit to TIGR
Genome
1,011
Significant signatures
2,886
Hits >1
184
No hit to TIGR genome
1,875
Unreliable signatures
6,448
Hits = 1
1,316
Hit to TIGR
Genome
1,587
Non significant signatures
3,562
Hits >1
271
No hit to TIGR genome
1,975
Hits = 1
37,838
Hit to TIGR
Genome
42,057
Significant signatures
76,915
Hits >1
4,219
No hit to TIGR genome
34,858
Reliable
Signatures
163,751
Hits = 1
1,313
Hit to TIGR
Genome
1,883
Non significant signatures
86,836
Hits >1
240
No hit to TIGR genome
84,953
Total number of distinct SBS signatures from 5 libraries
397,543
Hits = 1
1,803
Hit to TIGR
Genome
2,085
Significant signatures
8,684
Hits >1
282
No hit to TIGR genome
6,599
Unreliable signatures
233,792
Hits = 1
823
Hit to TIGR
Genome
950
Non significant signatures
225,108
Hits >1
127
No hit to TIGR genome
224,158
A.
B.
Additional File 1
