# Supplementary material for: Deep sequencing reveals the complex and coordinated transcriptional regulation of genes related to grain quality in rice cultivars
Source: BMC Genomics. 2011 Apr 14;12:190. doi: 10.1186/1471-2164-12-190 (PMC3098810; doi:10.1186/1471-2164-12-190)
Supplement: Additional file 2 — Classification of the MPSS and SBS signatures from the five libraries based on their location on the annotated gene (hits = 1) (See Meyers et al. 2004 [45]for details). [file 1471-2164-12-190-S2.DOC]

Additional File 2. Classification of the MPSS and SBS signatures from the five libraries based on their location on the annotated gene (hits=1) (See Meyers et al. 2004 for details)

| MPSS Signature category | Nipponbare (PSN) | | Cypress (PSC) | | LaGrue (PSL) | | Ilpumbyeo (PSI) | | YR15965 (PSY) | |
| --- | --- | --- | --- | --- | --- | --- | --- | --- | --- | --- |
|  | Total signatures | Grouped by gene (including transposons) | Total signatures | Grouped by gene (including transposons) | Total signatures | Grouped by gene (including transposons) | Total signatures | Grouped by gene (including transposons) | Total signatures | Grouped by gene (including transposons) |
| Class 1 (Exon, sense strand) | 4184 | 3778 | 3454 | 3171 | 4440 | 4014 | 4198 | 3821 | 3098 | 2893 |
| Class 2 (500 bp 3’-UTR) | 4950 | 4452 | 3852 | 3534 | 5396 | 4785 | 5373 | 4781 | 3887 | 3566 |
| Class 3 (Exon, antisense strand) | 569 | 526 | 329 | 314 | 591 | 552 | 519 | 484 | 340 | 314 |
| Class 4 (Un-annotated region) | 1,756 | 0 | 1,135 | 0 | 2,036 | 0 | 1,993 | 0 | 1,152 | 0 |
| Class 5 (Intron, sense strand) | 378 | 360 | 276 | 266 | 451 | 427 | 445 | 419 | 290 | 278 |
| Class 6 (Intron, antisense strand) | 101 | 98 | 69 | 66 | 130 | 121 | 120 | 117 | 73 | 72 |
| Class 7 (Span splice site, sense strand) | 138 | 137 | 110 | 109 | 154 | 152 | 140 | 140 | 104 | 104 |
| Classes 1,2,5,7 (Sense signatures) | 9650 | 7840 | 7692 | 6502 | 10441 | 8354 | 10156 | 8180 | 7379 | 6280 |
| Classes 3,6 (Antisense signatures) | 670 | 616 | 398 | 380 | 721 | 669 | 639 | 596 | 413 | 384 |
| Total | 10320 | 8085 | 8090 | 6672 | 11162 | 8610 | 10795 | 8407 | 7792 | 6436 |

| SBS Signature category | Nipponbare (PSN02) | | Cypress (PSC01) | | LaGrue (PSL01) | | Ilpumbyeo (PSI02) | | YR15965 (PSY02) | |
| --- | --- | --- | --- | --- | --- | --- | --- | --- | --- | --- |
|  | Total signatures | Grouped by gene (including transposons) | Total signatures | Grouped by gene (including transposons) | Total signatures | Grouped by gene (including transposons) | Total signatures | Grouped by gene (including transposons) | Total signatures | Grouped by gene (including transposons) |
| Class 1 (Exon, sense strand) | 4,915 | 4,480 | 4,092 | 3,733 | 2,648 | 2,513 | 3,008 | 2,808 | 3,444 | 3,216 |
| Class 2 (500 bp 3’-UTR) | 11,524 | 9,008 | 9,807 | 7,892 | 8,104 | 6,703 | 9,218 | 7,484 | 8,143 | 6,795 |
| Class 3 (Exon, antisense strand) | 4,472 | 3,687 | 3,265 | 2,787 | 2,382 | 2,083 | 3,105 | 2,658 | 2,400 | 2,087 |
| Class 4 (Un-annotated region) | 2521 | 0 | 1,976 | 0 | 1,576 | 0 | 1,596 | 0 | 1359 | 0 |
| Class 5 (Intron, sense strand) | 1,363 | 1,188 | 1,082 | 960 | 824 | 751 | 858 | 769 | 861 | 769 |
| Class 6 (Intron, antisense strand) | 454 | 419 | 353 | 324 | 303 | 280 | 281 | 258 | 247 | 235 |
| Class 7 (Span splice site, sense strand) | 156 | 156 | 129 | 128 | 85 | 85 | 95 | 94 | 112 | 111 |
| Classes 1,2,5,7 (Sense signatures) | 17,958 | 12,738 | 15,110 | 11,137 | 11,661 | 9,143 | 13,179 | 10,066 | 12,560 | 9,729 |
| Classes 3,6 (Antisense signatures) | 4,926 | 4,028 | 3,618 | 3,064 | 2,685 | 2,339 | 3,386 | 2,890 | 2,647 | 2,296 |
| Total | 25,405 | 13,337 | 20,704 | 11,622 | 15,922 | 9,631 | 18161 | 10,578 | 16,566 | 10,207 |
