# Supplementary material for: Deep sequencing reveals the complex and coordinated transcriptional regulation of genes related to grain quality in rice cultivars
Source: BMC Genomics. 2011 Apr 14;12:190. doi: 10.1186/1471-2164-12-190 (PMC3098810; doi:10.1186/1471-2164-12-190)
Supplement: Additional file 5 — Network of lysine and aspartate family amino acid biosynthesis and degradation. http://www.gramene.org. Only the genes with 5-fold up- or down-regulation in Cypress (PSC) or Ilpumbyeo (PSI) compared with that in LaGrue or YR15965 are shown. The positive number in parenthesis indicates up-regulation and the negative number in parenthesis indicates down-regulation. The first value in parenthesis shows the fold change in expression either in LaGrue or YR15965, and the second value shows the fold change in expression in Nipponbare. The italicized and underlined bold number before the parenthesis shows the MPSS/SBS signature class [45]. Green indicates that the gene was identified by SBS only. Red indicates that the gene was identified by MPSS only. Blue indicates that the gene was identified by both MPSS and SBS. [file 1471-2164-12-190-S5.PPT]

## Slide 1
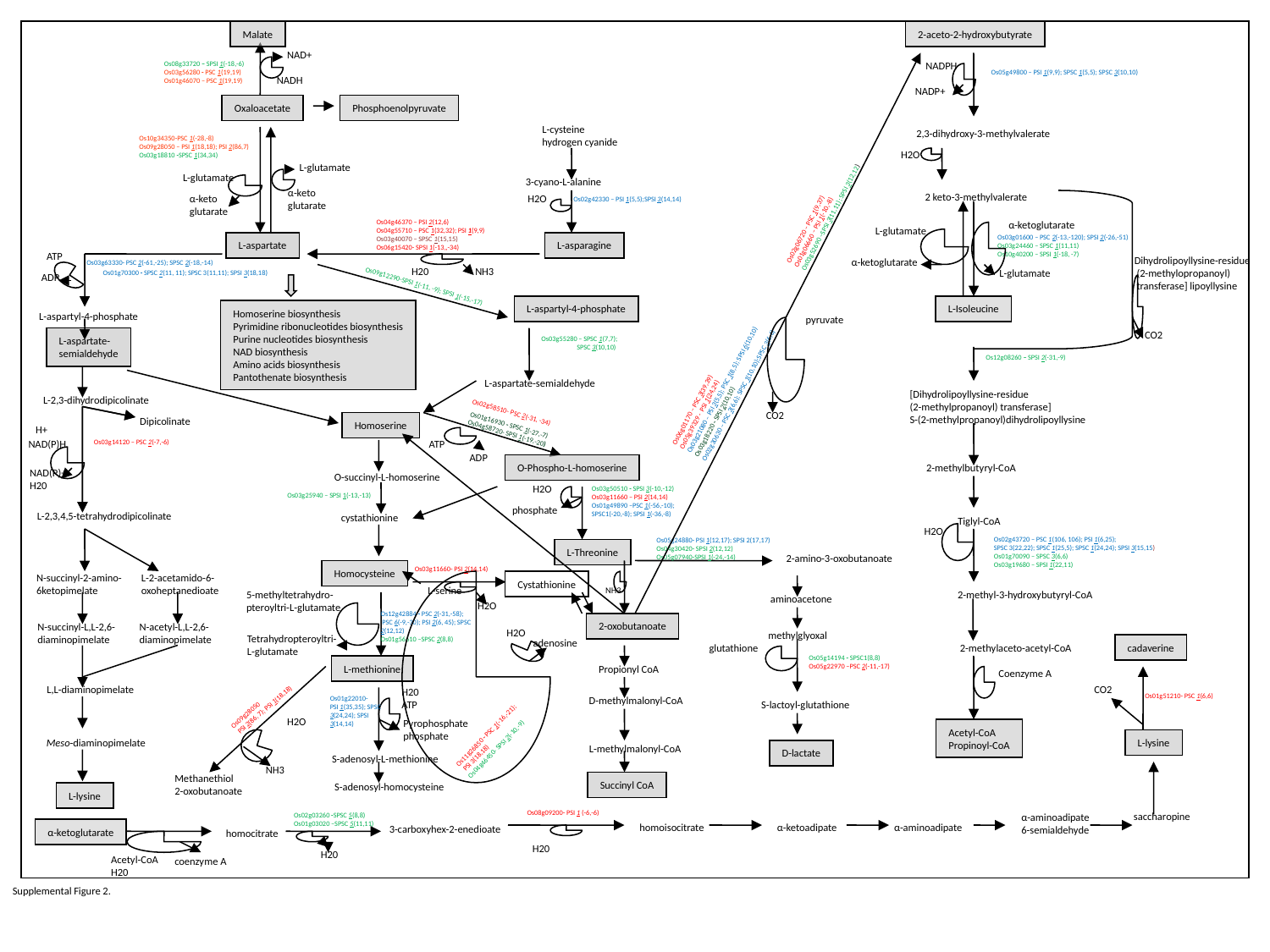

Malate
2-aceto-2-hydroxybutyrate
NAD+
Os08g33720 – SPSI 1(-18,-6)
Os03g56280 - PSC 1(19,19)
Os01g46070 – PSC 1(19,19)
NADPH
Os05g49800 – PSI 1(9,9); SPSC 1(5,5); SPSC 3(10,10)
NADH
NADP+
Oxaloacetate
Phosphoenolpyruvate
L-cysteine
hydrogen cyanide
2,3-dihydroxy-3-methylvalerate
Os10g34350-PSC 1(-28,-8)
Os09g28050 – PSI 1(18,18); PSI 2(86,7)
Os03g18810 -SPSC 1(34,34)
H2O
L-glutamate
L-glutamate
3-cyano-L-alanine
α-keto
glutarate
2 keto-3-methylvalerate
α-keto
glutarate
H2O
Os02g42330 – PSI 1(5,5);SPSI 3(14,14)
Os02g06720 – PSC 1(9,37)
Os01g06660 – PSI 1(-10,-8)
Os03g52690 –SPSI 3(11,11); SPSI 3(12,12)
Os04g46370 – PSI 2(12,6)
Os04g55710 – PSC 1(32,32); PSI 1(9,9)
Os03g40070 – SPSC 1(15,15)
Os06g15420- SPSI 1(-13,,-34)
α-ketoglutarate
L-glutamate
Os03g01600 – PSC 2(-13,-120); SPSI 2(-26,-51)
Os03g24460 – SPSC 1(11,11)
Os10g40200 – SPSI 1(-18, -7)
L-aspartate
L-asparagine
ATP
Dihydrolipoyllysine-residue
 (2-methylopropanoyl)
 transferase] lipoyllysine
α-ketoglutarate
Os03g63330- PSC 2(-61,-25); SPSC 2(-18,-14)
Os01g70300 - SPSC 2(11, 11); SPSC 3(11,11); SPSI 3(18,18)
H20
NH3
L-glutamate
ADP
Os09g12290-SPSI 1(-11, -9); SPSI 1(-15,-17)
L-aspartyl-4-phosphate
L-Isoleucine
Homoserine biosynthesis
Pyrimidine ribonucleotides biosynthesis
Purine nucleotides biosynthesis
NAD biosynthesis
Amino acids biosynthesis
Pantothenate biosynthesis
L-aspartyl-4-phosphate
pyruvate
CO2
L-aspartate-
semialdehyde
Os03g55280 – SPSC 1(7,7);
 SPSC 3(10,10)
Os06g01170 – PSC 3(39,39)
Os05g39329 - PSI 1(24,24)
Os03g21080 – PSI 3(5,5); PSC 1(8,5); SPSI 6(10,10)
Os03g18220 - SPSI 2(10,10)
Os02g30630 – PSC 3(6,6); SPSC 1(10,10);SPSC 3(6,6)
Os12g08260 – SPSI 2(-31,-9)
L-aspartate-semialdehyde
[Dihydrolipoyllysine-residue
(2-methylpropanoyl) transferase]
S-(2-methylpropanoyl)dihydrolipoyllysine
L-2,3-dihydrodipicolinate
Os02g58510- PSC 2(-31, -34)
Os01g16930 - SPSC 1(-27,-7)
Os04g58720- SPSI 1(-19,-20)
CO2
Dipicolinate
Homoserine
H+
ATP
NAD(P)H
Os03g14120 – PSC 2(-7,-6)
ADP
O-Phospho-L-homoserine
2-methylbutyryl-CoA
NAD(P)+
H20
O-succinyl-L-homoserine
H2O
Os03g50510 - SPSI 3(-10,-12)
Os03g11660 – PSI 2(14,14)
Os01g49890 –PSC 1(-56,-10);
SPSC1(-20,-8); SPSI 1(-36,-8)
Os03g25940 – SPSI 1(-13,-13)
phosphate
L-2,3,4,5-tetrahydrodipicolinate
cystathionine
Tiglyl-CoA
H2O
Os05g24880- PSI 1(12,17); SPSI 2(17,17)
Os04g30420- SPSI 2(12,12)
Os05g07940-SPSI 1(-24,-14)
Os02g43720 – PSC 1(106, 106); PSI 1(6,25);
SPSC 3(22,22); SPSC 1(25,5); SPSC 1(24,24); SPSI 3(15,15)
Os01g70090 – SPSC 3(6,6)
Os03g19680 – SPSI 1(22,11)
L-Threonine
2-amino-3-oxobutanoate
Homocysteine
Os03g11660- PSI 2(14,14)
N-succinyl-2-amino-
6ketopimelate
L-2-acetamido-6-
oxoheptanedioate
Cystathionine
L-serine
NH3
5-methyltetrahydro-
pteroyltri-L-glutamate
2-methyl-3-hydroxybutyryl-CoA
aminoacetone
H2O
Os12g42884 - PSC 2(-31,-58);
 PSC 6(-9,-30); PSI 2(6, 45); SPSC 2(12,12)
Os01g56610 –SPSC 2(8,8)
N-succinyl-L,L-2,6-
diaminopimelate
N-acetyl-L,L-2,6-
diaminopimelate
2-oxobutanoate
H2O
methylglyoxal
Tetrahydropteroyltri-
L-glutamate
adenosine
glutathione
2-methylaceto-acetyl-CoA
cadaverine
Os05g14194 - SPSC1(8,8)
Os05g22970 –PSC 2(-11,-17)
L-methionine
Propionyl CoA
Coenzyme A
L,L-diaminopimelate
CO2
H20
ATP
Os01g22010-
PSI 1(35,35); SPSC 3(24,24); SPSI 3(14,14)
D-methylmalonyl-CoA
Os01g51210- PSC 1(6,6)
Os09g28050
 PSI 2(86, 7); PSI 1(18,18)
S-lactoyl-glutathione
H2O
Pyrophosphate
phosphate
Os11g26850 - PSC 1(-16,-21);
 PSI 3(18,18)
Os04g46450- SPSI 2(-10,-9)
Acetyl-CoA
Propinoyl-CoA
Meso-diaminopimelate
L-lysine
L-methylmalonyl-CoA
D-lactate
S-adenosyl-L-methionine
NH3
Methanethiol
2-oxobutanoate
Succinyl CoA
S-adenosyl-homocysteine
L-lysine
Os02g03260 -SPSC 5(8,8)
Os01g03020 –SPSC 5(11,11)
Os08g09200- PSI 1 (-6,-6)
α-aminoadipate
6-semialdehyde
saccharopine
homoisocitrate
α-ketoadipate
α-aminoadipate
3-carboxyhex-2-enedioate
α-ketoglutarate
homocitrate
H20
H20
Acetyl-CoA
H20
coenzyme A
Supplemental Figure 2.
