# Supplementary material for: Deep sequencing reveals the complex and coordinated transcriptional regulation of genes related to grain quality in rice cultivars
Source: BMC Genomics. 2011 Apr 14;12:190. doi: 10.1186/1471-2164-12-190 (PMC3098810; doi:10.1186/1471-2164-12-190)
Supplement: Additional file 7 — Conserved cis elements in the promoter region of the highly induced genes (≥50 fold) in Cypress (compared to LaGrue and Nipponbare) and Ilpumbyeo (compared to YR15965 and Nipponbare) that are involved in seed development. [file 1471-2164-12-190-S7.DOC]

Additional File 7. Conserved *cis* elements in the promoter region of the highly induced genes (≥50 fold) in Cypress (compared to LaGrue and Nipponbare) and Ilpumbyeo (compared to YR15965 and Nipponbare) that are involved in seed development

| **Sl.**  **No** | **Motif name** | ***cis* element** | **Function** | **Reference** |
| --- | --- | --- | --- | --- |
| 1 | CAATBOX1 | CAAT | Seed specificity, storage protein, legumin, seed development | [46] |
| 2 | GTGANTG10 | GTGA | Tobacco pollen gene | [47] |
| 3 | WRKY71OS | TGAC | Transcriptional repressor of GA signaling in rice aleurone cells. | [48] |
| 4 | GATABOX | GATA | Endosperm development, seed germination | [49] |
| 5 | EBOXBNNAPA | CANNTG | Storage protein genes | [50] |
| 6 | POLLEN1LELAT52 | AGAAA | Pollen development | [51] |
| 7 | POLASIG1 | AATAAA | Seed germination | [52] |
| 8 | SEF4MOTIFGM7S | RTTTTTR | Soyabean developing seeds' embryo development | [53] |
| 9 | CGACGOSAMY3 | CGACG | Amylase expression during sugar starvation | [54] |
| 10 | WBOXHVISO1 | TGACT | Regulatory transcription factor in starch synthesis | [55] |
| 11 | ABRELATERD1 | ACGTG | Seed germination | [56] |
| 12 | -300ELEMENT | TGHAAARK | Upstream regions of glutenin, alpha and gamma gliadin genes | [57] |
| 13 | CAREOSREP1 | CAACTC | Rice seed germination | [58] |
| 14 | CANBNNAPA | CNAACAC | Upstream regions of seed storage protein genes | [59] |
| 15 | AMYBOX1 | TAACARA | Upstream regions of alpha-amylase gene | [60] |
| 16 | AACACOREOSGLUB1 | AACAAAC | Endosperm specific gene expression | [61] |
| 17 | BOXIIPCCHS | ACGTGGC | Seed germination | [62] |
| 18 | 2SSEEDPROTBANAPA | CAAACAC | Upsteram regions of storage protein genes | [63] |
| 19 | ACGTABOX | TACGTA | Seed development | [64] |
| 20 | AMYBOX2 | TATCCAT | Upstream regions of alpha amylase genes in rice | [65] |
| 21 | -300CORE | TGTAAAG | Endosperm specific gene expression | [66] |
| 22 | ACGTCBOX | GACGTC | Seed development | [67] |
| 23 | ACGTOSGLUB1 | GTACGTG | Endosperm specific gene expression | [68] |
| 24 | CEREGLUBOX2PSLEGA | TGAAAACT | Cereal glutenin gene control element | [69] |
| 25 | GADOWNAT | ACGTGTC | Seed germination | [70] |
